# Wechsler Intelligence Scale for Children-Revised Profiles in Children with Attention Deficit Hyperactivity Disorder: Impact of Comorbid Autistic Traits

**DOI:** 10.5152/eurasianjmed.2026.261449

**Published:** 2026-09-25

**Authors:** Buket Kılıç, Dilek Ünal

**Affiliations:** 1 Department of Child and Adolescent Psychiatry, Hacettepe University, Ankara, Türkiye

**Keywords:** Attention deficit hyperactivity disorder, autistic traits, Wechsler intelligence scale for children-revised

## Abstract

**Background:**

This study aimed to identify cognitive differences between children diagnosed with attention deficit hyperactivity disorder (ADHD) with and without comorbid autistic traits by using the Wechsler Intelligence Scale for Children-Revised (WISC-R).

**Methods:**

This retrospective study evaluated children aged 8-16 years diagnosed with ADHD according to Diagnostic and Statistical Manual of Mental Disorders, Fifth Edition criteria. Participants were stratified based on their Social Responsiveness Scale scores to assess autistic traits. Exclusion criteria comprised autism spectrum disorder, intellectual disability, schizophrenia, bipolar disorder, anxiety, depression, and a Full-Scale IQ below 70. Cognitive profiles were assessed using the WISC-R. To control for Type I error across the 12 WISC-R variables, a Bonferroni correction adjusted the statistical significance threshold to *P* < .004.

**Results:**

The cohort included 16 children with autistic traits and 20 without. Only the WISC-R Block Design subtest revealed a significant difference, with lower scores observed in the group with autistic traits (*P* = .003). No significant differences were found in full-Scale, verbal, or performance IQ scores.

**Conclusion:**

The significant difference observed exclusively in the Block Design subtest suggests that comorbid autistic traits in children with ADHD may specifically impair visual–spatial and motor-integrated skills. Future studies incorporating objective motor assessments are warranted to further elucidate these cognitive relationships.

**Main Points**

- Children with attention deficit hyperactivity disorder (ADHD) and comorbid autistic traits exhibited significantly lower scores on the Block Design subtest, suggesting underlying challenges in visual-motor integration and spatial processing during structured tasks.
- No statistically significant differences were observed between the 2 groups regarding full-scale, verbal, or performance IQ scores.
- Larger-scale studies utilizing contemporary intelligence measures are warranted to further clarify the cognitive profiles of children with ADHD and comorbid autistic traits.

## Introduction

Attention deficit hyperactivity disorder (ADHD) is a childhood-onset neurodevelopmental disorder characterized primarily by inattention, hyperactivity, and impulsivity.^1^ Although a unique cognitive profile for ADHD remains elusive, affected individuals routinely display impairments across multiple neuropsychological domains. Specifically, executive dysfunctions—such as deficits in response inhibition, vigilance, working memory, and planning—are widely documented.^2^ Moreover, the widespread heterogeneity in these cognitive profiles suggests that cognitive performance does not strictly mirror the clinical presentation or progression of symptoms.^3^

Attention deficit hyperactivity disorder and autism spectrum disorder (ASD) exhibit overlapping impairments in attention and executive functioning. Autistic traits comprise subthreshold social, communicative, and repetitive behavioral deficits that fall below the diagnostic threshold for ASD in the general population.^4^ Occurring in roughly 30% of children with ADHD, the presence of these traits often leads to more complex clinical phenotypes.^4^^,^^5^

Cognitive profiles for distinct, fully diagnosed ADHD and ASD are extensively reported in the literature, yet data become inconsistent at the intersection of these 2 disorders. Studies on high-functioning ASD populations often show underperformance in comprehension, object assembly, coding, and symbol search, alongside strengths in information, similarities, and block design.^6^^-^^10^ However, when autistic traits are embedded within an ADHD diagnosis, comparative research suggests these children score lower on full-scale IQ, digit span, block design, and digit symbol subtests compared to ADHD peers without these traits.^11^^-^^13^ The present study aims to address this gap, as empirical data remain scarce regarding children with ADHD who exhibit subthreshold autistic traits without fulfilling the complete criteria for ASD.

The study tested the following hypotheses:

- First, it was hypothesized that the ADHD group with autistic traits would score lower on the comprehension, object assembly, and digit symbol subtests, but higher on the information, similarities, and block design subtests compared to the group without these traits.
- Second, it was anticipated that the group with autistic traits would exhibit a significantly lower full-scale IQ than their peers without autistic traits.

## Material and Methods

### Participants

The study sample included children aged 8-16 years with Diagnostic and Statistical Manual of Mental Disorders, Fifth Edition (DSM)-5-defined ADHD (APA, 2013). This retrospective chart review evaluated patients presenting to the Hacettepe University Department of Child and Adolescent Psychiatry between May and December 2022. Participants were grouped using Social Responsiveness Scale (SRS) scores, where scores ≥ 60 indicated the presence of autistic traits and scores < 60 indicated their absence. Although the SRS is inherently a dimensional screening instrument that evaluates autistic traits along a continuum, participants in this study were categorized into distinct groups based on specific established cut-off scores to facilitate statistical analysis. However, these groups were conceptualized as representing levels of “autistic trait severity” rather than establishing formal clinical diagnoses.^14^^-^^15^

Strict exclusion criteria maintained sample homogeneity. Individuals with diagnoses of ASD, intellectual disability, schizophrenia, bipolar disorder, or a WISC-R full-Scale IQ below 70 were excluded. Additionally, based on the schedule for affective disorders and schizophrenia (K-SADS) interviews, patients with comorbid anxiety or major depressive disorders were excluded to prevent confounding effects on timed cognitive subtests. To avoid treatment-induced cognitive alterations, only treatment-naïve participants without prior psychotropic medication or formal psychotherapy history were eligible.

The analysis of this retrospective archival data was approved by the Hacettepe University Health Sciences Research Ethics Committee on November 8, 2024 (Ref: SBA 24/930). Legal guardians had previously provided informed consent for all clinical evaluations.

### Materials

### Schedule for Affective Disorders and Schizophrenia for School-Aged Children-Present and Lifetime Version

Originally developed by Kaufman et al, this semi-structured interview evaluates a broad spectrum of psychiatric disorders by integrating data from the child, parents, and clinical observations.^16^ The Turkish version has demonstrated high reliability and validity in alignment with DSM-5 criteria.^17^ The K-SADS was utilized to confirm ADHD diagnoses and screen for comorbid psychiatric conditions.

### Wechsler Intelligence Scale for Children-Revised

The WISC-R was administered by a certified clinical psychologist to evaluate participants’ cognitive profiles.^18^ The scale yields 3 primary composite scores: verbal IQ, performance IQ, and full-scale IQ (mean = 100, SD = 15). It encompasses various subtests, including information, similarities, block design, and object assembly (mean scaled score = 10, SD = 3). The Turkish adaptation of the scale has been previously validated for clinical use.^19^

### Social Responsiveness Scale

The SRS, a 65-item parent-report questionnaire, was used to quantify the severity of autistic symptoms in social communication and repetitive behaviors.^14^ Total T-scores of 60 or above (≥ 60) serve as the clinical threshold for identifying significant autistic traits.^15^ The Turkish version of the SRS has exhibited strong psychometric properties, including a Cronbach’s α of 0.86 and robust test-retest reliability.^20^

### Statistical Analysis

Statistical analyses were performed using IBM SPSS Statistics for Windows, Version 23.0 (IBM Corp.; Armonk, NY, USA). Given the small sample size, continuous variables were analyzed using the Mann–Whitney *U*-test, while categorical data were assessed via Pearson’s chi-square or Fisher’s exact test. The initial significance level was set at *P* < .05. To account for the inflated Type I error rate across the 12 WISC-R variables, a Bonferroni correction adjusted the significance threshold to *P* < .004. Effect sizes were computed for all comparisons to determine clinical relevance. This study did not use AI and/or LLM tools during the preparation of this manuscript.

## Results

The sample comprised 36 participants: 16 children with autistic traits and 20 without. Demographic and clinical profiles—including age, gender distribution, parental education, and socioeconomic status—showed no statistically significant differences between the groups. Additionally, both groups were comparable regarding ADHD presentation types and the prevalence of comorbid specific learning disorder (SLD), ensuring a well-balanced baseline (Table 1).

Evaluating WISC-R scores across groups yielded a significant difference in only 1 subtest. Children with autistic traits scored significantly lower on the block design subtest compared to those without (*Z* = −2.974, *P* = .003), with a substantial effect size (*r* = 0.502) indicating clinical significance. In contrast, no significant disparities were observed in broader intelligence indices, including full-scale IQ, verbal IQ, and performance IQ. Similarly, the remaining WISC-R subtests showed no statistically significant differences between the 2 groups (Table 2).

The percentages given in the table are column percentages. The median (interquartile range) is shown for the age variable to which the Mann–Whitney *U*-test was applied. The chi-squared test was applied to the other variables.

## Discussion

The primary objective of this study was to explore whether comorbid autistic traits alter the cognitive profile of children with ADHD. Based on the literature, the hypothesis states that the autistic traits group would underperform on the comprehension subtest due to Theory of Mind difficulties; on object assembly due to weak central coherence; and on digit symbol due to impairments in processing speed, visual-motor integration, and working memory. The hypothesis also states that full-scale IQ to be lower, reflecting cumulative executive deficits, particularly in cognitive flexibility.^13^^,^^21^^,^^22^ Conversely, the hypothesis states that they would demonstrate superior performance on the information subtest owing to crystallized intelligence and long-term memory retrieval; on the similarities subtest due to a propensity for systematic thinking; and on the block design subtest driven by strengths in visual-spatial analysis and local information processing styles.^7^^,^^9^ Yet, contrary to expectations, the findings revealed no statistically significant variations between ADHD children with and without autistic traits in terms of broad IQ indices or most WISC-R subtests.

An examination of prior research on children with ADHD and comorbid autistic traits reveals both parallel and divergent outcomes. For example, Kotte et al^13^ found that the autistic traits group scored significantly lower on full-scale IQ, the freedom from distractibility index, block design, and digit symbol subtests than the non-traits ADHD group. Likewise, a study by Cooper et al^11^ using the WISC-III or WISC-IV indicated that lower full-scale IQ and reduced working memory index scores correlated with autistic traits in ADHD. Additionally, a 10-year prospective follow-up study utilizing the WISC-III/IV or WAIS-III showed that individuals with autistic traits underperformed on the arithmetic subtest and the freedom from distractibility index at the end of the follow-up period.^12^ Overall, these investigations imply that comorbid autistic traits in ADHD typically predict lower total IQ scores and deficits in working memory-related measures, which authors often link to underlying challenges in executive functions and cognitive flexibility.^13^

A key explanation for the unique findings could be the reliance on the WISC-R. Although the WISC-R is a highly validated and historically robust instrument, it lacks the refined index structures—such as fluid reasoning or working memory—available in contemporary editions like the WISC-IV and WISC-V.^23^^,^^24^ This reliance on an older cognitive profile may have reduced the sensitivity to detect subtle, fractional cognitive discrepancies that contemporary tools might capture. Nonetheless, studies utilizing more recent scales to investigate children with ADHD and autistic traits who do not meet full ASD criteria remain sparse.^12^^,^^13^ Consequently, a substantial gap persists in the literature, emphasizing the need for further research in this specific domain.

Alternatively, 1 plausible explanation for the lack of differentiation across verbal and performance domains is the “ceiling effect” of executive dysfunction inherent to ADHD. Core deficits in working memory, processing speed, and cognitive flexibility are already severely pronounced in children with ADHD, largely driving their general test-taking performance.^2^^,^^25^ Consequently, the incremental presence of subthreshold autistic traits may not further lower these specific subtest scores on the WISC-R. In this regard, incorporating a non-ADHD control group into the study design could have provided a clearer understanding of this overlapping cognitive impact.

The only cognitive domain that differentiated the 2 groups was the block design subtest, where children with ADHD and comorbid autistic traits exhibited significantly lower scores. Interestingly, better performance had been predicted for the autistic traits group for this subtest; yet, contrary to the expectations, performance was significantly worse. This divergent result may stem from limitations such as the small sample size, the study’s retrospective design, and the utilization of the WISC-R—an instrument that is no longer fully contemporary.

While strong performance on block design is frequently highlighted in high-functioning ASD populations,^6^^,^^8^^,^^10^ and even among general population cohorts with elevated autistic traits, the literature remains highly limited.^26^ Because past research has predominantly focused on categorical ASD diagnoses rather than dimensional traits, the precise effect of these traits on cognitive profiles remains elusive. Therefore, the finding might not be an exception. Supporting this notion, Kotte et al^13^ also found that children with ADHD presenting with autistic traits achieved lower block design scores than those without such traits.

The block design subtest evaluates visual-spatial processing, organizational skills, and non-verbal problem-solving. Being a time-limited task, its performance is also inherently influenced by motor proficiency.^27^ Studies show that children with autistic traits frequently experience motor difficulties and clumsiness.^11^^,^^13^ Similarly, Gillberg described children with ADHD who present with DAMP (deficits in attention, motor control, and perception) syndrome, noting that two-thirds of those with severe profiles also present with ASD features.^28^^,^^29^ Consequently, the reduced block design scores in the autistic traits group might stem from these shared motor coordination challenges.

Moreover, autistic traits might further amplify the visual-spatial deficits, inhibitory control problems, and working memory impairments already present in ADHD, leading to poorer test outcomes.^30^ However, the interpretation regarding this specific mechanism must remain cautious, as the study did not utilize objective, gold-standard neuro-motor assessment batteries.^31^^,^^32^ To fully elucidate how autistic traits influence various neurodevelopmental phenotypes, future research must integrate current intelligence scales with contemporary executive function and neuro-motor assessments.

Several factors limit the generalizability of the findings. First, the small sample size led to limited subgroup sizes, potentially restricting the statistical power to detect subtle cognitive variations. Second, the lack of a healthy control group precludes evaluating how autistic traits interact with typical neurodevelopment. Third, relying on the older WISC-R rather than contemporary instruments like the WISC-V may alter the characterization of the cognitive profiles.^24^ Fourth, group stratification was based on the SRS, a dimensional screening scale rather than a formal diagnostic tool. Although clinical evaluations and the K-SADS successfully ruled out full ASD diagnoses, incorporating gold-standard structured diagnostic instruments like the ADOS or ADI-R would have enhanced the internal validity and phenotypic precision of the subgroups.^33^^,^^34^ Finally, the retrospective nature of this study carries inherent limitations regarding data completeness and confounding variables, preventing the determination of definitive causal relationships.

In conclusion, this study contributes to the sparse literature comparing intelligence profiles in children with ADHD with and without dimensional autistic traits. The findings suggest a specific vulnerability in the block design subtest among those with elevated autistic traits. These insights underscore the clinical importance of evaluating dimensional neurodevelopmental phenotypes during routine cognitive assessments. Future research utilizing contemporary intelligence scales, objective neuro-motor batteries, and larger cohorts is required to further elucidate the complex cognitive architecture within the ADHD-autism overlap.

## Figures and Tables

**Table 1.** Comparison of Sociodemographic and Clinical Characteristics of ADHD Diagnostic Groups With and Without Autistic Traits

	Autistic Traits (+) n = 16	Autistic Traits (−) n = 20	Test Statistic	*P*
Age	124.5 (47.00)	122 (35.25)	−0.573	.566
Sex Female (%) Male (%)	9 (56.2) 7 (43.8)	8 (40.0) 12 (60.0)	0.403	.503
Mother’s education level High school and above (%) Below high school (%)	12 (75) 4 (25)	13 (65) 7 (35)	0.419	.718
Father’s education level High school and above (%) Below high school (%)	9 (56.2) 7 (43.8)	16 (80) 4 (20)	2.363	.159
Socioeconomic level Medium and above (%) Below medium (%)	15 (93.8) 1 (6.2)	17 (85) 3 (15)	0.689	.613
ADHD clinical presentation Attention deficit predominant (%) Combined (%)	5 (31.2) 11 (68.8)	8 (40) 12 (60)	0.038	.846
Specific learning disorder None (%) There is (%)	9 (56.2) 7 (43.8)	15 (75) 5 (25)	0.689	.406

ADHD, attention deficit hyperactivity disorder.

The percentages given in the table are column percentages. The median (interquartile range) is shown for the age variable to which the Mann–Whitney *U*-test was applied. The chi-squared test was applied to the other variables.

**Table 2.** Comparison of WISC-R Results of ADHD Diagnostic Groups With and Without Autistic Traits

WISC-R Results	Autism Traits (+) n = 16	Autism Traits (−) n = 20	Test Statistic	*P*	Effect Size
Total IQ	92.50 (17.25)	98.00 (20.00)	−0.653	.514	0.108
Verbal IQ	91.50 (14.75)	89.50 (21.25)	−0.096	.924	0.016
Performance IQ	95.00 (11.25)	100 (19.75)	−1.244	.213	0.207
Information	6.00 (5.00)	7.50 (3.75)	−0.908	.364	0.153
Similarities	10.00 (5.00)	10.50 (5.25)	−0.603	.546	0.101
Arithmetic	9.00 (3.00)	9.50 (3.75)	−0.692	.489	0.116
Comprehension	10.00 (4.00)	8.50 (5.00)	−0.572	.567	0.096
Picture completion	10.00 (2.00)	10.50 (4.75)	−0.405	.686	0.068
Picture arrangement	9.00 (4.00)	8.00 (2.00)	−1.213	.225	0.205
Block design	9.00 (2.00)	12.00 (6.00)	−2.974	.003*	0.502
Object assembly	10.00 (3.00)	11.50 (4.75)	−1.544	.123	0.260
Digit symbol	9.00 (4.00)	11.00 (5.75)	−0.419	.675	0.070

The Mann–Whitney *U*-test was applied, and the median (interquartile range) is shown.

ADHD, attention deficit hyperactivity disorder; WISC-R, Wechsler intelligence scale for children-revised.

*Significant after Bonferroni correction (adjusted threshold: *P* < .004).

## Data Availability

The data that support the findings of this study are available on request from the corresponding author.
